# Supplementary material for: The Effect of Formulation Variables on the Manufacturability of Clopidogrel Tablets via Fluidized Hot-Melt Granulation—From the Lab Scale to the Pilot Scale
Source: Pharmaceutics. 2024 Mar 13;16(3):391. doi: 10.3390/pharmaceutics16030391 (PMC10975944; doi:10.3390/pharmaceutics16030391)
Supplement: Supplementary file 1 [file pharmaceutics-16-00391-s001.zip › pharmaceutics-2869610-supplementary.pdf]

pharmaceutics

MDPI

Supplementary Material S1

Table S1. Database used for multivariate data analysis.

Primary ID	Class ID	Granulation temperature (°C)	Granulation time (min)	Macrogol type	Kolliwax content (%)	Loss on drying (%)	Bulk density (g/100 mL)	Flow-out time (sec/100 g)	Particle size distribution, wt.% retained on sieve (µm)						Compression force, core (kN)	Resistance to crushing, core (N)	Disintegration time, core (sec)	Resistance to crushing, fct. (N)	Disintegration time, fct. (sec)
									> 1000	800	400	200	80	< 80					
**L1**	ED	50	5	PEG8000P	These batches were not lubrified	0.33	This set-point resulted in batch failure								Batches were not compressed to tablets				
**L2**	ED	65	5	PEG8000P		0.53	58.6	14	22.7	9.9	24.2	24.9	18	0.4					
**L3**	ED	50	30	PEG8000P		0.46	57.2	-	0.0	0	1.7	14.2	40.1	42.8					
**L4**	ED	65	30	PEG8000P		0.40	54.2	13	0.4	7.2	37.3	42.1	11.8	0.4					
**L5**	ED	50	5	PEG6000P		0.23	58.2	-	0.2	0.1	1.8	16.1	38.4	42.6					
**L6**	ED	65	5	PEG6000P		0.47	53.6	13	0.6	7.1	36	36.3	19.5	0.5					
**L7**	ED	50	30	PEG6000P		0.23	59.5	42	0.2	0.2	2.2	23	40.1	33.6					
**L8**	ED	65	30	PEG6000P		0.43	54.4	13	1.9	4.2	25.2	36.8	31.1	0.7					
**L9**	ED	50	5	PEG8000C		0.46	58.7	11	15.4	9.2	24.9	20	28.8	1.7					
**L10**	ED	65	5	PEG8000C		0.43	57.2	-	0.5	0.8	5.7	12.1	38.8	41.5					
**L11**	ED	50	30	PEG8000C		0.53	55.1	-	0.0	0.2	1.5	23.1	34.8	39.7					
**L12**	ED	65	30	PEG8000C		0.50	57.1	-	0.1	0.2	1.6	16.9	41.4	39.4					
**L13**	ED	57.5	17.5	PEG8000P		0.50	52.3	13	2.6	5.2	42.2	45.3	4.6	0.5					
**L14**	ED	57.5	17.5	PEG8000P		0.40	56.9	-	0.2	0.2	1.7	20.6	40.8	36					
**L15**	ED	57.5	17.5	PEG8000P		0.17	56.1	-	0.0	0.1	1.4	12.8	40.1	44.7					
**L16**	ED-L	65	10	PEG8000P	1.67	0.44	51.6	13	0.5	4.2	23.3	43.4	26.6	1.2	16.5	117	977	128	943
**L17**	ED-L	65	10	PEG6000P	1.67	0.44	54.2	13	0.2	5.6	33.2	36.4	22.8	0.8	16.5	113	690	126	882
**L18**	ED-L	65	10	PEG8000P	1.33	0.34	53.3	13	0.2	2.4	21	44.4	29.8	1.2	16.5	130	732	132	874
**L19**	ED-L	65	10	PEG8000P	2.00	0.34	53.3	13	0.2	2.4	21	44.4	29.8	1.2	16.5	127	1092	120	1227

**L20**	ED-L	65	10	PEG6000P	1.33	0.42	54.1	13	0.2	6.4	29.6	36.4	25.8	0	16.5	120	877	116	621
**L21**	ED-L	65	10	PEG6000P	1.33	0.4	51.6	13	0.4	3	20.6	46.2	27.2	1.6	15.5	124	878	124	979
**L22**	ED-L	65	10	PEG6000P	1.33	0.36	55.3	12	0.2	5.2	26.6	40.6	25.8	1.4	15.5	117	654	126	932
**L23**	ED-L	65	10	PEG6000P	1.33	0.36	54	12	0.2	3.3	27.2	33.4	30.4	2	15.5	119	873	124	1011
**L24**	ED-L	65	10	PEG8000P	1.33	0.32	52.7	12	0.4	3.6	22.4	38.4	32.2	1.2	16	120	683	127	673
**L25**	ED-L	65	10	PEG6000P	1.33	0.48	56.6	12	0.6	9.8	29.8	29.6	29.2	1.8	16	113	751	121	776
**P1.1**	P	65	10	PEG6000P	1.33	0.36	58	12	2.2	7.7	25.4	22.2	38.7	2.7	10	109	630	This batch was not film-coated	
**P1.2**	P	65	10	PEG6000P	1.33	0.36	58	12	2.2	7.7	25.4	22.2	38.7	2.7	12	116	630		
**P1.3**	P	65	10	PEG6000P	1.33	0.36	58	12	2.2	7.7	25.4	22.2	38.7	2.7	14	125	630		
**P1.4**	P	65	10	PEG6000P	1.33	0.36	58	12	2.2	7.7	25.4	22.2	38.7	2.7	16	124	720		
**P1.5**	P	65	10	PEG6000P	1.33	0.36	58	12	2.2	7.7	25.4	22.2	38.7	2.7	18	129	720		
**P1.6**	P	65	10	PEG6000P	1.33	0.36	58	12	2.2	7.7	25.4	22.2	38.7	2.7	20	127	750		
**P2.1**	P	65	10	PEG6000P	1.33	0.44	66	13	0.0	3.7	24.6	19	47.7	4.3	15	136	Inappropriate tablet compression		
**P2.2**	P	65	10	PEG6000P	1.33	0.44	66	13	0.0	3.7	24.6	19	47.7	4.3	16	139			
**P2.3**	P-DIS	65	10	PEG6000P	1.33	0.44	66	13	0.0	3.7	24.6	19	47.7	4.3	18	138	720	148	900
**P2.4**	P-DIS	65	10	PEG6000P	1.33	0.44	66	13	0.0	3.7	24.6	19	47.7	4.3	20	147	810	146	900
**P3.1**	P	60	10	PEG8000P	1.33	0.52	59.6	20	0.0	0	7.3	26.8	43.2	20.8	16	116	690		
**P3.2**	P-DIS	60	10	PEG8000P	1.33	0.52	59.6	20	0.0	0	7.3	26.8	43.2	20.8	18	124	720	130	810
**P3.3**	P-DIS	60	10	PEG8000P	1.33	0.52	59.6	20	0.0	0	7.3	26.8	43.2	20.8	20	125	720	137	810
**P3.4**	P	60	10	PEG8000P	1.33	0.52	59.6	20	0.0	0	7.3	26.8	43.2	20.8	22	118	720	These samples were not film-coated	
**P3.5**	P	60	10	PEG8000P	1.33	0.52	59.6	20	0.0	0	7.3	26.8	43.2	20.8	24	123	720		
**P4.1**	P-DIS	60	10	PEG6000P	1.33	0.58	63.6	19	0.0	0	14.6	16.2	52.7	15.7	18	123	780	138	1020
**P4.2**	P-DIS	60	10	PEG6000P	1.33	0.58	63.6	19	0.0	0	14.6	16.2	52.7	15.7	20	125	780	135	1020
**P5**	P-DIS	60	10	PEG8000P	1.33	0.63	60	13	0.0	0	21.5	23.6	43.2	10.8	20	134	720	139	900
**P6**	P-DIS	60	10	PEG8000P	1.33	0.58	60	12	0.0	3.7	25.2	24.1	41.7	4.8	20	139	720	139	900
**P7**	P-DIS	60	10	PEG8000P	1.33	0.64	60	12	0.0	3.7	23.7	22.8	42.4	6.8	20	137	720	147	900

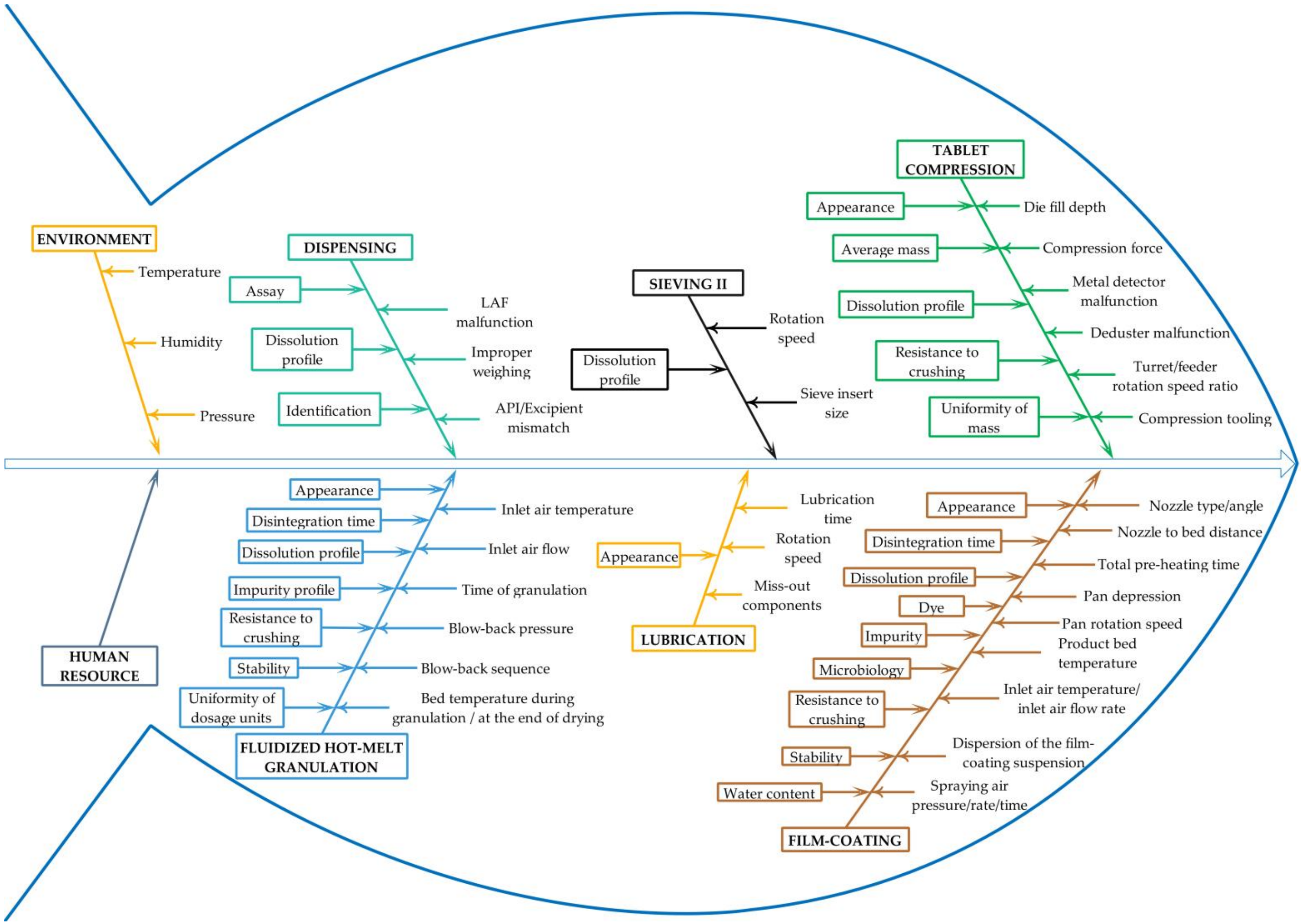

Supplementary Figure S1. Ishikawa Chart presenting the most important factors taken into consideration that might influence product performance.

pharmaceutics

MDPI

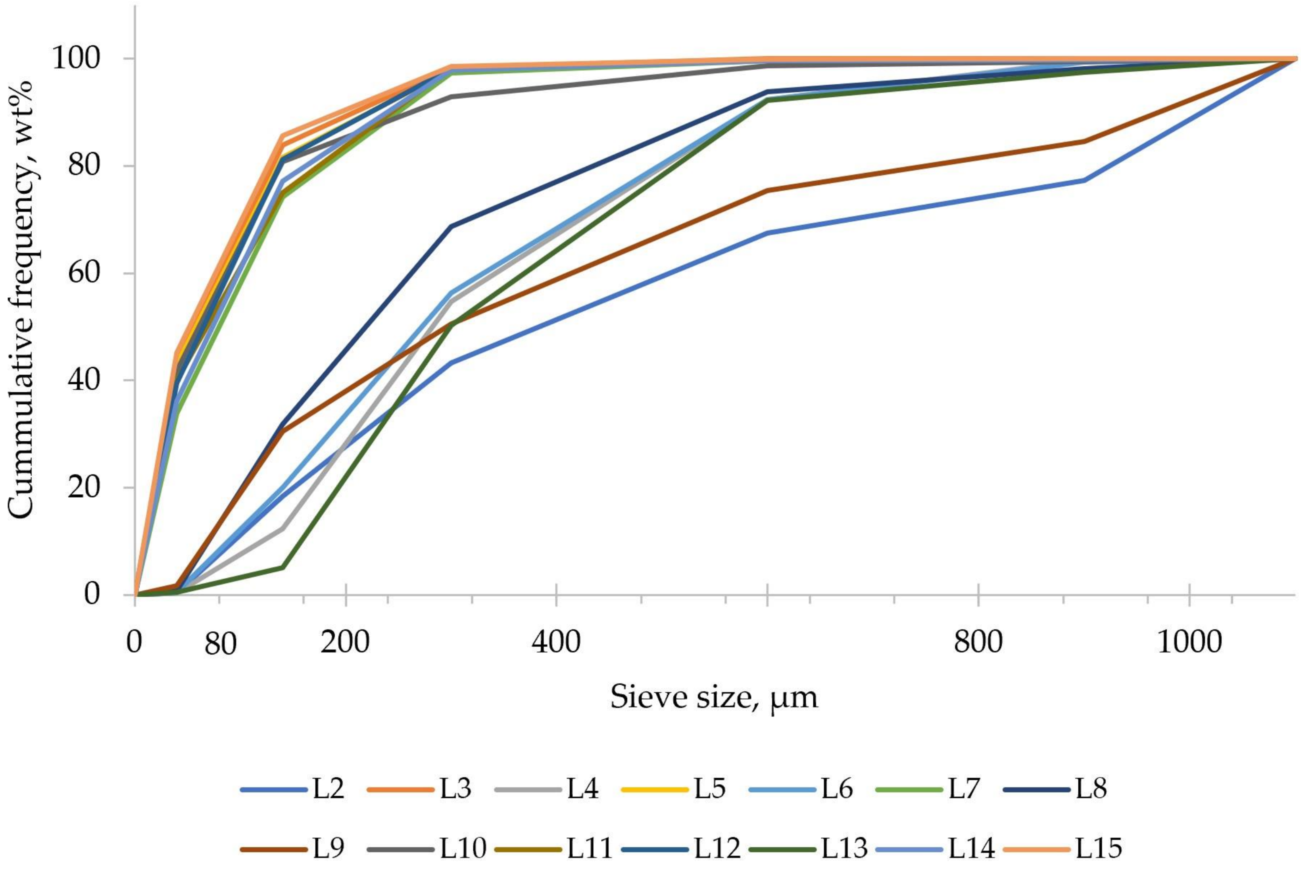

Supplementary Figure S2. Granulometric distribution of laboratory scale batches

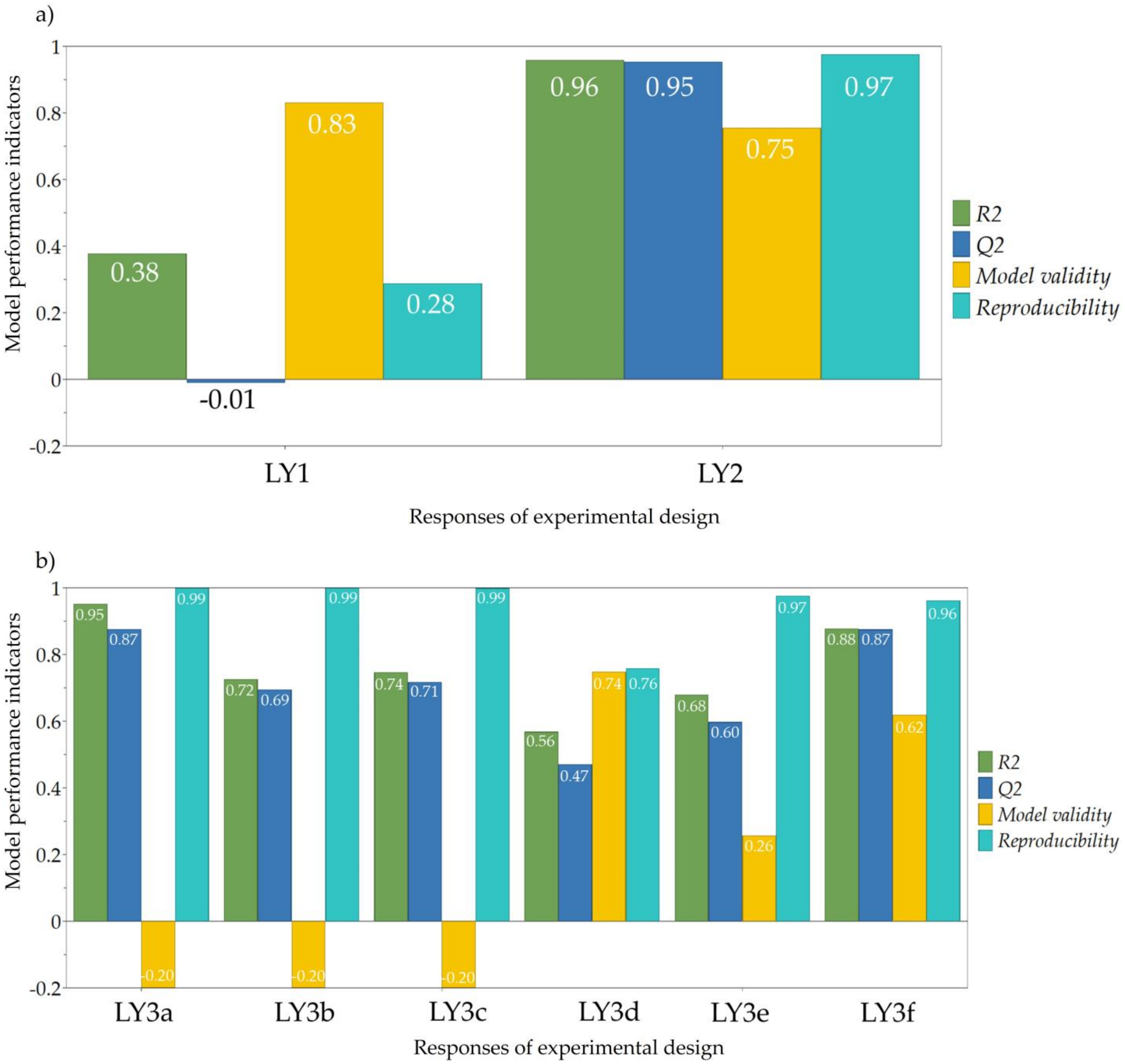

Supplementary Figure S3. Illustration of the product performance indicators for a) bulk density (LY1), flow-out time (LY2) and b) particle size distribution (LY3a-LY3b)

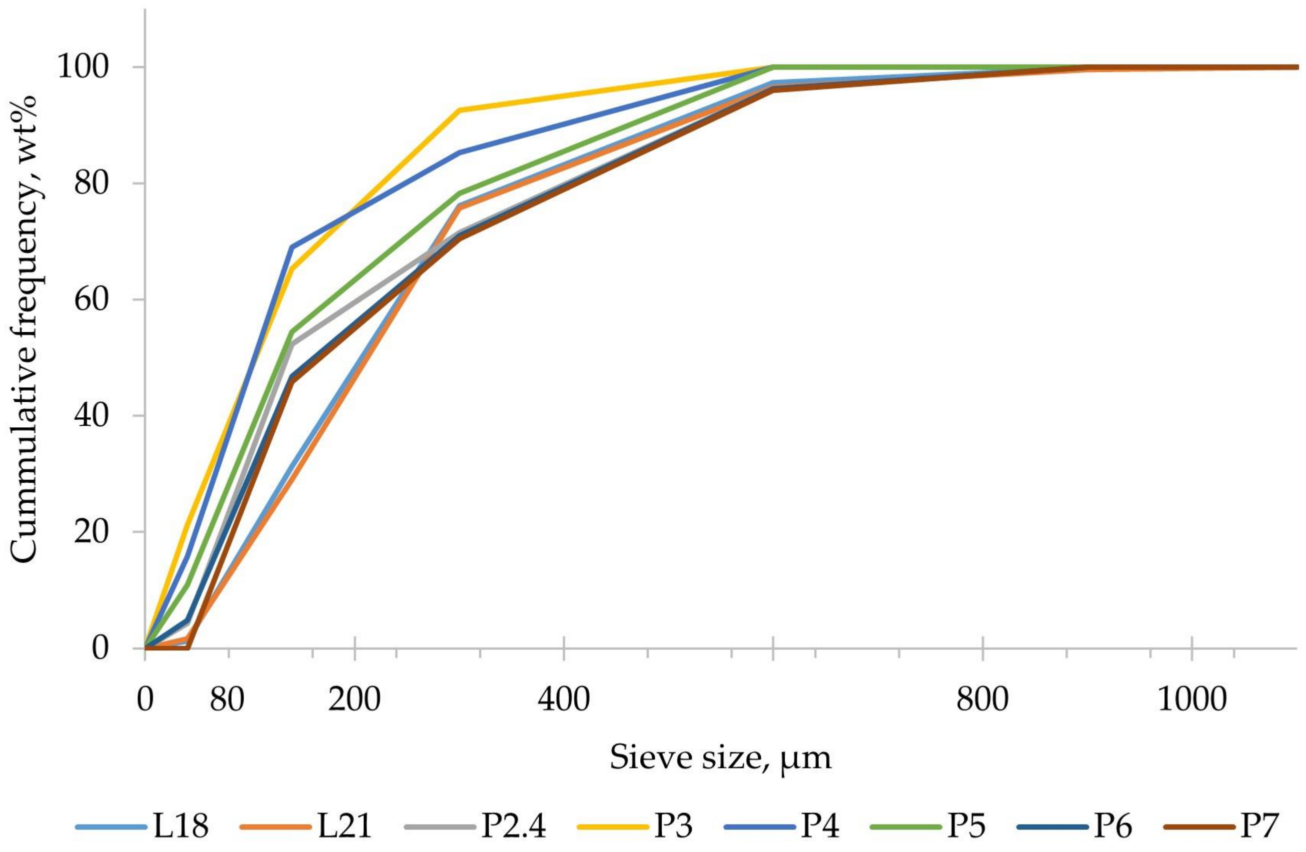

Supplementary Figure S4. Granulometric distribution of laboratory vs. pilot scale batches

pharmaceutics MDPI

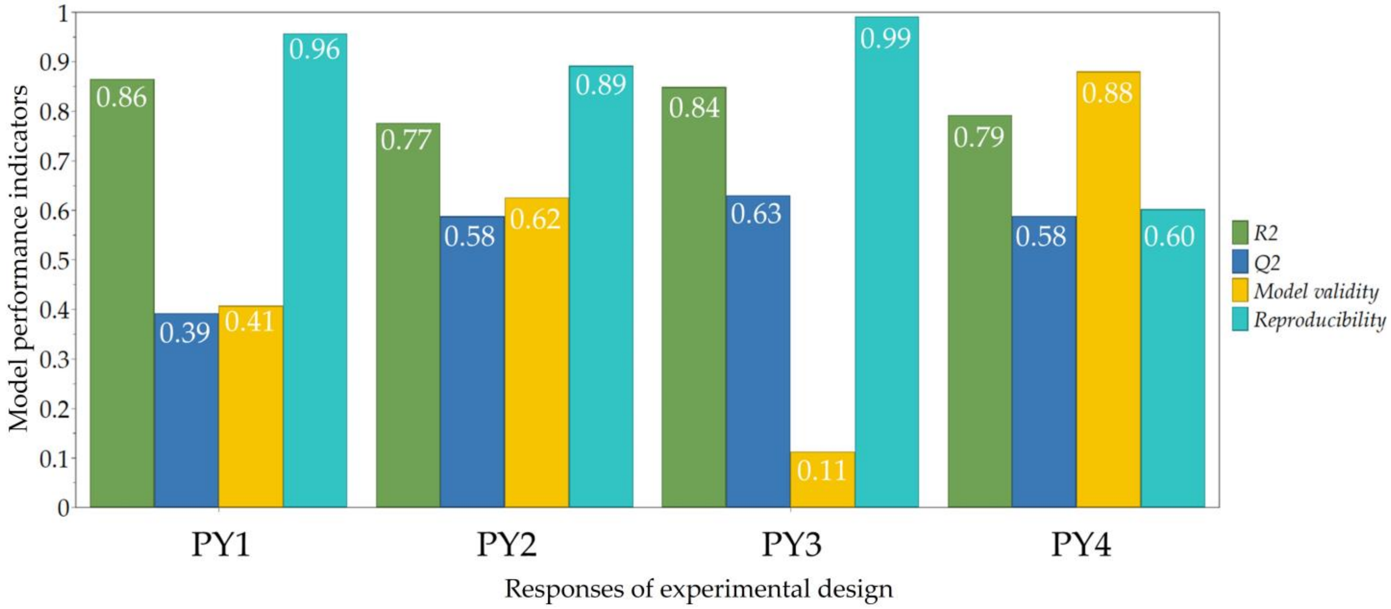

Supplementary Figure S5. Illustration of model performance indicators in case of experimental design for tablet compression. PY1 – Disintegration time, PY2 – Cpk, PY3 – Resistance to crushing, PY4 - Friability
